# Comparative Transcriptome and Metabolome Analyses of Broccoli Germplasms with Purple and Green Curds Reveal the Structural Genes and Transitional Regulators Regulating Color Formation

**DOI:** 10.3390/ijms24076115

**Published:** 2023-03-24

**Authors:** Shaozhe Wen, Ning Li, Shuhui Song, Ning Liu, Yunhua Ding

**Affiliations:** 1 Beijing Vegetable Research Center (National Engineering Research Center for Vegetables), Beijing Academy of Agriculture and Forestry Science, Beijing 100097, China; 2 Key Laboratory of Biology and Genetic Improvement of Horticultural Crops (North China), Ministry of Agriculture and Rural Affairs, Beijing 100097, China; 3 Key Laboratory of Urban Agriculture (North China), Ministry of Agriculture and Rural Affairs, Beijing 100097, China; 4 Institute of Agri-food Processing and Nutrition, Beijing Academy of Agriculture and Forestry Sciences, Beijing 100097, China; 5 Beijing Key Laboratory of Vegetable Germplasm Improvement, Beijing 100097, China

**Keywords:** broccoli, anthocyanin biosynthesis, transcriptome, metabolome, gene expression

## Abstract

Owing to the high anthocyanin content, broccoli varieties with purple curds have become more popular in food inventories, while the genetic mechanisms of anthocyanin biosynthesis pathways remain largely unknown. We bred a pair of near-isogenic lines (NILs), GB767 and PB767, whose curds exhibited green and purple colors, respectively, due to the purple sepals of florets. RNA sequencing and widely targeted metabolic analyses were conducted. Compared with GB767, eighteen anthocyanin biosynthesis-related genes exhibited significantly higher expressions in PB767, and in turn, the expression level of *BolMYBL2.1* was attenuated. A comparison of the metabolites in the flavonoid biosynthetic pathways revealed 142 differentially accumulated metabolites, among which higher content of anthocyanins was responsible for the purple color of PB767. Interestingly, the total cyanidin contents were similar between the curds of NILs, whereas total delphinidin contents were increased by more than 170 times in purple curds, presumably due to a non-canonical *F3′H*/*CYP75B* gene, *BolC02g015480.2J*, with elevated expression in PB767. Furthermore, correlation analysis further confirmed that the identified nineteen DEGs were significantly correlated with seven differentially accumulated anthocyanins in PB767. Together, these results identified the metabolic factors and genes that contribute to the purplish curds, which could lay foundations for the breeding programs of purple broccoli.

## 1. Introduction

Broccoli (*Brassica oleracea* L. var. *italica*) is a nutritious vegetable crop, and its curds are rich in provitamin A (β-carotene), vitamin C (ascorbate), vitamin E (tocopherol), and other health-promoting phytochemicals such as phenolics, flavonoids, and glucosinolates [1,2,3]. Purple broccoli cultivars are known for the high amounts of anthocyanins in their curds [4]. Anthocyanins are a group of water-soluble, flavonoid pigments [5,6]. There are three major anthocyanins, pelargonidin, cyanidin, and delphinidin, all of which to a large extent contribute to the red, orange, or violet colors of plant organs [7,8,9]. In addition to their roles in vegetative and floral color development, anthocyanins can protect plants from biotic attack and abiotic stress [10]. More importantly, the intake of anthocyanins can reduce the incidence of cardiovascular disease, cancer, and hypertension [11,12], which makes purple broccoli an eye-catching vegetable in the current commercial market.

The molecular pathway of anthocyanin biosynthesis has been intensively investigated in the model plant *Arabidopsis*, and to varying degrees in non-model plant species such as maize, petunia, tobacco, and so on [13]. The anthocyanin biosynthesis pathway is highly conserved across plant species and is generally divided into three major steps: 4-coumarate-CoA biosynthesis, flavonoid biosynthesis, and anthocyanin biosynthesis [13,14]. The first step involves phenylalanine ammonia-lyase (PAL), cinnamate 4-hydroxylase (C4H), and 4-coumarate-CoA ligase (4CL). In the second step, dihydroflavonols are formed by the catalyzation of chalcone synthase (CHS), chalcone isomerase (CHI), flavanone 3-hydroxylase (F3H), and flavonoid 3′,5′-hydroxylase (F3′5′H). Finally, dihydroflavonol 4-reductase (DFR), anthocyanin synthase (ANS), and UDP-glucose:flavonoid 3-O-glucosyltransferase (UFGT) sequentially catalyze dihydroflavonols into anthocyanins. 

A number of transcription factors (TFs) have been found to act as regulators of the anthocyanin biosynthesis pathways in model and crop plants [15]. The early biosynthesis genes (EBGs) including *CHS*, *CHI,* and *F3H* are directly activated by R2R3-MYB transcription factors such as MYB11, MYB12, and MYB111, whereas the late biosynthesis genes (LBGs) including *F3′H*, *F3′5′H*, *DFR*, *ANS,* and *UFGT* are modulated by the MYB-bHLH-WD40 (MBW) complex in *Arabidopsis* [16]. These MYB regulators or MBW complexes often upregulate the expressions of anthocyanin biosynthesis genes by binding to their promoters. In *Arabidopsis*, the MYB transcription factor *AtTT2* combined with factors *AtTT8* (bHLH42) and *AtTTG1* (WD40) form an MBW complex, which promotes the expression of the proanthocyanidin biosynthesis core gene *BANYULS* to pigment the seed coat [17]. However, some MYB TFs such as *AtMYB12* and *VvMYBF1* can positively regulate flavonoid biosynthesis without the involvement of bHLH and WD40 cofactors [18,19]. In addition, several MYB transcription factors, such as *AtMYBL2*, *FaMYB1*, *VvMYB4*, and *MdMYB16/17/111*, competing with R2R3-MYB positive regulators for binding to bHLH proteins, can inhibit the expressions of anthocyanin biosynthesis genes [20,21,22,23]. In *Brassica* crops, several R2R3-MYB transcription factors were also involved in the regulation of anthocyanin biosynthesis in the leaf or flower organs. For example, *BrMYB2* and *BnaA07.PAP2* positively regulate the anthocyanin biosynthesis in the purple head of Chinese cabbage (*B. rapa* ssp. *pekinensis* L.) and the flowers of *B. napus*, respectively [24,25]. Nevertheless, *BoMYBL2-1* negatively regulates anthocyanin biosynthesis in purple cabbage (*B. oleracea* L. var. *capitata*) [26].

During the process of the purple broccoli breeding program, we identified a pair of near-isogenic lines (NILs) with contrasting curd colors. GB767 produces green curds whereas PB767 has purplish curds. Further observations suggested that the color difference between the NILs occurred in the sepals of florets. Here, the two NILs were used as experimental materials for the transcriptome and metabolome analysis to investigate how the anthocyanin biosynthesis pathways are changed in purple broccoli. The differentially expressed genes involved in regulating anthocyanin biosynthesis and differentially produced metabolites belonging to anthocyanin were identified and a further correlation analysis was performed. The results of this study will help to deepen the understanding of anthocyanin biosynthesis in broccoli and provide valuable theoretical support for the breeding of purple broccoli.

## 2. Results

### 2.1. Phenotypic Difference of Curd Color between GB767 and PB767

**Figure 1:**
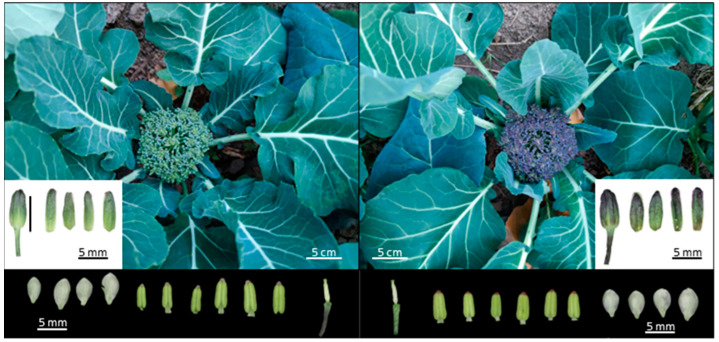
Phenotypic characteristics of GB767 (**left**) and PB767 (**right**) broccoli NILs. The florets were further dissected to compare their color differences between different floral organs. Photos of flower buds and their sepals of GB767 and PB767 are shown on the bottom left and right, respectively. Photos of the petals, stamens pistils, styles, and pedicels of two NILs are shown below.

GB767 and PB767 are a pair of near-isogenic lines of broccoli isolated from a breeding intermediate material with contrasting colors of curds. They have similar agronomic traits except for the difference in curd color. As shown in Figure 1, visual inspections suggested that the PB767 curds exhibited deeper blue-purple pigmentations, especially during the early stage of curd development, whereas GB767 curds showed green colors. With the aid of a stereo microscope, we confirmed that the color difference between GB767 and PB767 is mainly reflected on the sepals of florets as well as the pedicels; however, the colors of petals, stamens, pistils, and styles are indistinguishable between the NILs. Therefore, the sepals of GB767 and PB767 were selected for further transcriptome and metabolome analysis.

### 2.2. Transcriptome Sequencing and Data Quality

To identify the candidate genes that cause the color difference of broccoli curds, transcriptome sequencing and the clean reads blast to the reference genome Braol_JZS_V2.0 (http://brassicadb.cn/#/Download/ (accessed on 9 December 2022)) of six samples (GB767-1, GB767-2, GB767-3, PB767-1, PB767-2, and PB767-3) from the sepals of flower buds for a pair of near-isogenic lines under the same conditions were performed. A total of about 40.68 Gb clean bases were obtained by RNA-seq after quality control of the raw data, with an average of 6.78 Gb for each sample. The percentage range of Q30 values was 93.83–94.41%. The content of GC ranged from 47.07 to 47.73% (Table 1). The data indexes of these reads indicated that the sequencing quality is reliable and suitable for further analysis.

**Figure 2:**
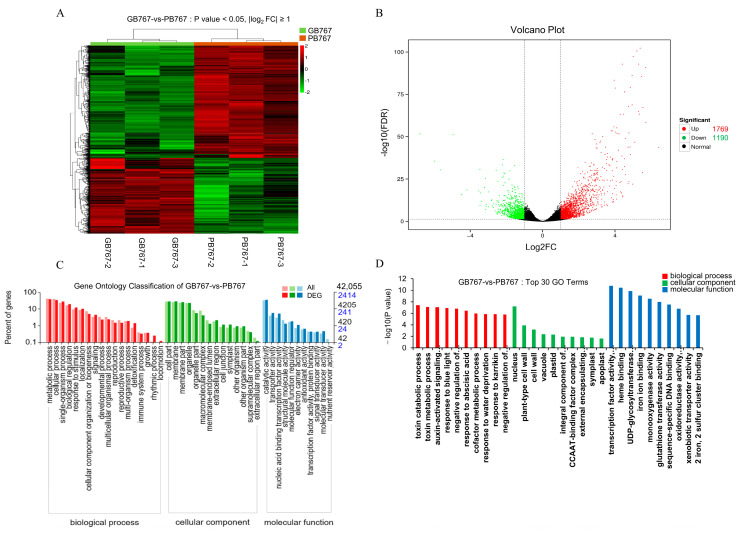
Identification and GO functional enrichment analysis of DEGs in GB767 vs. PB767. (**A**) Heatmap and hierarchical clustering of DEGs. (**B**) Volcano plot showing the number of upregulated and downregulated genes. (**C**) Comparison of the distribution of DEGs and all expressed genes at GO level 2. (**D**) Top 30 terms of GO functional enrichment analysis.

Subsequently, the clean reads of each sample were mapped to the cabbage JZS genome. The ratio of mapped reads to clean reads varied from 85.39–88.05% (GB767) and 85.24–89.15% (PB767), respectively. Among them, 80.23–83.63% (GB767) and 80.37–85.44% (PB767) were uniquely mapped (Table S1). The mRNA expression level was calculated using FPKM (fragments per kilobase of transcript per million mapped reads) to further compare the expressions of different genes among different samples. A box plot and density graph of the mRNA expression level are shown in Figure S1. The principal component analysis confirmed the genetic differences in gene expression between the pair of near-isogenic lines of broccoli (Figures S2 and S3). The clustering heatmap of expression levels showed that the six samples were divided into two categories (Figure 2A).

### 2.3. GO and KEGG Term Classification of Differentially Expressed Genes (DEGs) in GB767 vs. PB767

Differentially expressed genes (DEGs) were screened under the false discovery rate (FDR) ≤ 0.05 and |log2foldchange| ≥ 1 and compared to 8 public databases (GO, KEGG, NR, Swiss-Prot, Pfam, COG, KOG, and eggNOG) to determine their potential functions (Table S2). A total of 2959 DEGs were identified, and the volcano map showed that there were 1769 upregulated and 1190 downregulated DEGs in the purple curd lines (PB767) compared with the green curd lines (GB767) (Figure 2B). Among them, 2414 DEGs were annotated by 2411 GO terms and divided into biological process, cellular component, and molecular function (Figure 2C). Subsequently, GO enrichment analysis showed that the top 30 GO terms with the most significant enrichment included “Phenylalanine ammonia-lyase activity” (GO:0045548), “Vacuole” (GO:0005773), “Transcription factor activity, sequence-specific DNA binding” (GO:0003700), “UDP-glycosyltransferase activity” (GO:0008194), “Glutathione transferase activity” (GO:0004364), and so on (Figure 2D). As expected, the anthocyanidin-related pathways were significantly enriched in the PB767 groups. The results showed that anthocyanidin biosynthesis pathways were implicated in the occurrence of purple sepals in broccoli.

**Figure 3:**
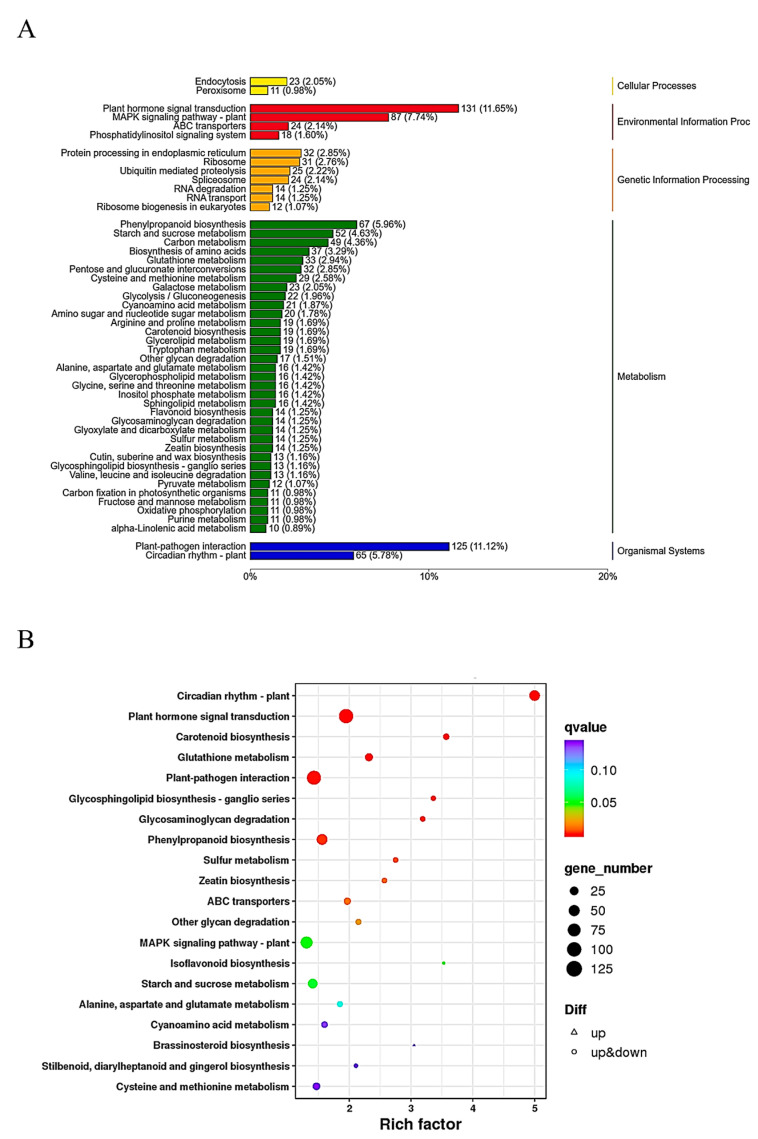
KEGG pathway enrichment analysis of DEGs in GB767 vs. PB767. (**A**) KEGG classification of DEGs. (**B**) The bubble diagram of the top 20 enriched KEGG pathways. The larger the bubble size, the more the number of DEGs. The smaller the q value, the greater the significance.

KEGG functional enrichment analysis was also conducted to clarify the roles of these DEGs in GB767 vs. PB767 (Figure 3A). A total of 733 DEGs were classified into the metabolism category that involved 35 metabolic pathways, which was the classification with the largest number of DEGs. Some of the 35 metabolic pathways, such as “Phenylpropanoid biosynthesis” (ko00940), “Glutathione metabolism” (ko00480), and “Isoflavonoid biosynthesis” (ko00943) were enriched and related to anthocyanin biosynthesis (Figure 3B). The result also indicated that alternated anthocyanidin biosynthesis could contribute to the purplish curds of PB767.

### 2.4. Transcription Factors and Anthocyanin-Related Genes in DEGs

Transcription factors (TFs) played important roles in the regulation of anthocyanin biosynthesis. In this study, 145 of the DEGs were annotated as having transcription factor activity in GO functional annotation, belonging to WRKY, AP2/ERF-ERF, bHLH, bZIP, C2H2, and other families (Table S3). In addition, according to the BRAD database and the study [24], 87 anthocyanin-related genes in Chinese cabbage were collected and used to compare the transcript levels of their homologous genes between GB767 and PB767 (Table S4). Meanwhile, 19 of them were DEGs between GB767 and PB767, and 18 of these DEGs were upregulated, including 4 *PAL* (*BolC04g011300.2J*, *BolC04g062040.2J*, *BolC08g037230.2J*, and *BolC04g038920.2J*), 3 *C4H* (*BolC04g019470.2J*, *BolC04g056700.2J*, and *BolC04g056710.2J*), 1 *CHS* (*BolC03g006710.2J*), 1 *F3′H* (*BolC09g064000.2J*), 1 *DFR* (*BolC01g003480.2J*), 1 *UFGT* (*BolC02g007850.2J*), 1 *AT* (*BolC08g048130.2J*), 3 *UGT* (*BolC07g045500.2J*, *BolC05g041090.2J,* and *BolC01g041020.2J*), 2 *MYB* (*BolC03g028310.2J* and *BolC06g037480.2J*), and *1 LBD* (*BolC09g010060.2J*), while 1 *MYB* (*BolC06g042490.2J*) was downregulated. Among them, absolute values of log2foldchange of *F3′H*, *3 UGT*, and *LBD* all exceeded 2, which indicated that these structural genes and TFs may play important roles in differences of anthocyanin biosynthesis and accumulation between GB767 and PB767, and are worthy of studying for their relationship.

### 2.5. Promoter Sequence Variation Analysis of 19 Differentially Expressed Anthocyanidin-Related Genes 

To study the relationship between differential expression and promoter sequence variation of 19 anthocyanin-related genes, we analyzed the sequence variation of 2kb promoter regions of these genes in GB767 and PB767. Among the 19 anthocyanin-related genes, only the promoters of *CHS2* (*BolC03g006710.2J*), *F3′H* (*BolC09g064000.2J*), and *LBD37.3* (*BolC09g010060.2J*) showed sequence variations between GB767 and PB767 (Table S5). We further used PlantCARE (http://bioinformatics.psb.ugent.be/webtools/plantcare/html/ (accessed on 9 December 2022)) to investigate whether the sequence changes could occur in cis-acting elements that might lead to expression alternations. The results showed that these sequence variations might have no effects on the expression effect in the promoter of GB767 or PB767; however, more experiments are needed to evaluate their effects on these genes. 

### 2.6. qRT-PCR Validation

**Figure 4:**
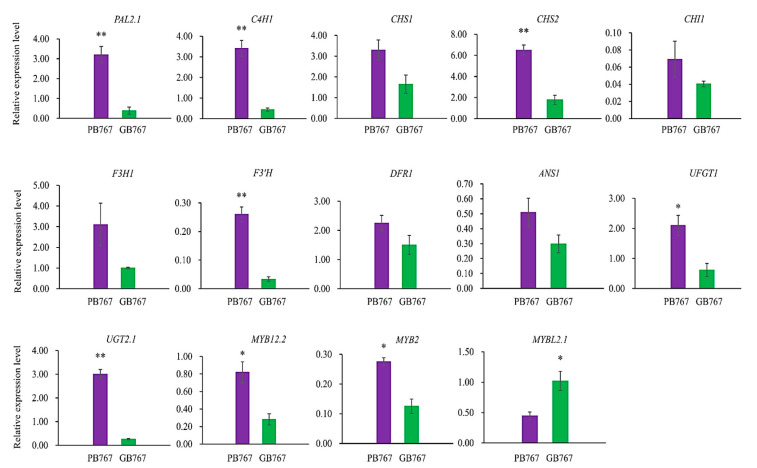
Expression levels of 14 anthocyanin-related genes between GB767 and PB767 by qRT-PCR. The green rectangular bar represents GB767 and the purple rectangular bar represents PB767. The value of the expression level was mean ± standard deviation (SD). * and ** represent significant differences, *p* < 0.05 and *p* < 0.01, respectively.

To validate the RNA-seq results, qRT-PCR analysis was performed on the 14 key anthocyanin-related genes involved in the three stages of anthocyanin biosynthesis, including 11 structural genes, *PAL2.1* (*BolC08g037230.2J*), *C4H1* (*BolC04g019470.2J*), *CHS1* (*BolC09g059140.2J*), *CHS2* (*BolC03g006710.2J*), *CHI1* (*BolC08g039120.2J*), *F3H1* (*BolC08g035550.2J*), *F3′H* (*BolC09g064000.2J*), *DFR1* (*BolC09g023150.2J*), *ANS1* (*BolC01g016390.2J*), *UFGT1* (*BolC02g007850.2J*), *UGT2.1* (*BolC05g041090.2J*), and 3 TFs, *MYB12.2* (*BolC03g028310.2J*), *MYB2* (*BolC06g037480.2J*), and *MYBL2.1* (*BolC06g042490.2J*). As shown in Figure 4, the expression trends of these genes were generally consistent with the RNA-seq results.

### 2.7. Analysis of the Widely Targeted Detection of Flavonoid Metabolome Data

**Figure 5:**
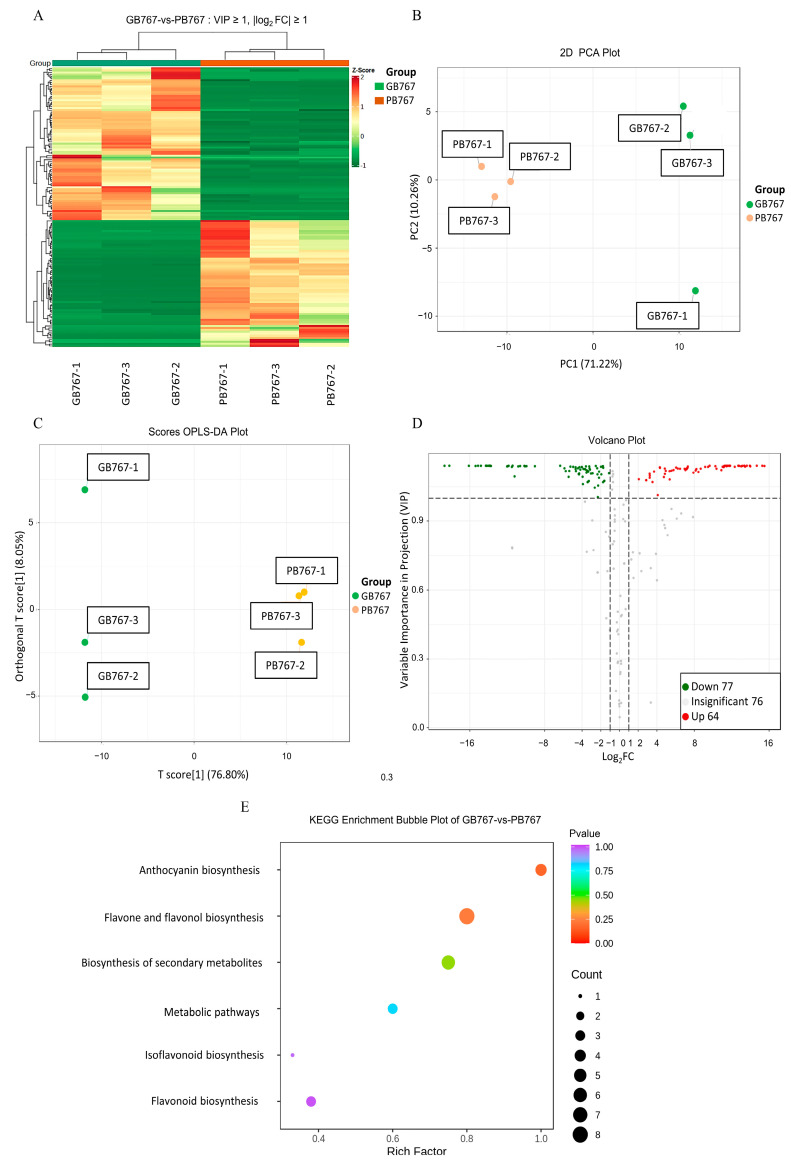
Identification and KEGG pathway enrichment analysis of DPMs in GB767 vs. PB767. (**A**) Heatmap and hierarchical clustering of DPMs. (**B**) PCA plot of the relative content of GB767 and PB767 metabolites. (**C**) OPLS-DA plot. (**D**) Volcano plot of upregulated and downregulated metabolites. (**E**) KEGG pathway enrichment bubble plot of DPMs.

To determine differences in types and relative contents of anthocyanins between GB767 and PB767, the widely targeted detection of flavonoid metabolome was conducted by UPLC-MS/MS. First, a total of 217 metabolites were identified in GB767 and PB767, 210 of them were flavonoids, and the remaining seven were tannins (Table S6). The cluster heatmap, PCA, and OPLS-DA plots showed differences in metabolites between GB767 and PB767 (Figure 5A–C). The volcano plot showed that 142 differentially produced metabolites (DPMs) based on an absolute log2foldchange ≥ 1 and VIP value ≥ 1 were filtered, 65 were upregulated, and 77 were downregulated (Figure 5D). Additionally, 16 DPMs were annotated to 6 anthocyanin-related pathways, including “Anthocyanin biosynthesis” (ko00942), “Flavone and flavonol biosynthesis” (ko00944), “Biosynthesis of secondary metabolites” (ko01110), “Metabolic pathways” (ko01100), “Isoflavonoid biosynthesis” (ko00943), and “Flavonoid biosynthesis” (ko00941) (Figure 5E, Table 2). A total of 7 of 142 DPMs were modified anthocyanins, with 3 belonging to the cyanidin type, 2 belonging to the delphinidin type, and 2 respectively belonging to the petunidin type and pelargonidin type (Table 3). Among them, the cyanidin-3-O-sophorotrioside, delphinidin-3-O-glucoside, delphinidin-3-O-galactoside, and petunidin-3-O-glucoside showed a high relative content in PB767 than GB767, whereas the cyanidin-3-O-glucoside, cyanidin-3-O-galactoside, and pelargonidin-3-O-glucoside, in contrast, have a high relative content in GB767 compared with PB767 (Table 3). Meanwhile, the relative content of the seven anthocyanins in PB767 was three times higher than that of GB767, which was consistent with the color difference between GB767 and PB767. According to the types and relative contents of the identified anthocyanins, we inferred that there are three typical branches of anthocyanin biosynthesis in broccoli, and substrates were more catalyzed to form cyanidin-type, pelargonidin-type, and delphinidin-type derivatives.

### 2.8. Correlation Analysis on Genes and Metabolites Involved in Anthocyanin Biosynthesis of GB767 and PB767

**Figure 6:**
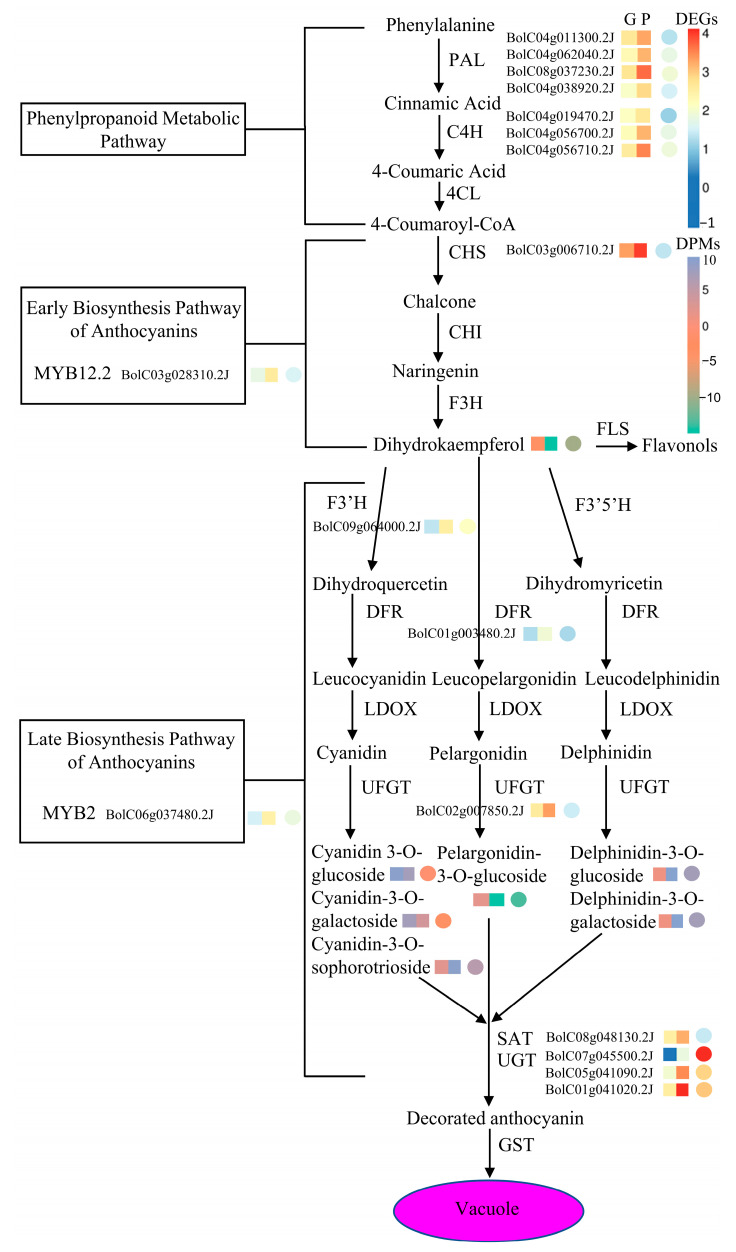
Genes and metabolites involved in anthocyanin biosynthesis in broccoli. The color scale next to the genes and metabolites indicates significant differences in their expression levels (FPKM) and relative contents (RC), respectively. The square color scale represents log10 FPKM of genes or log10 RC of metabolites and the circle color scale represents the log2foldchange of genes or metabolites.

In this study, we constructed a pathway map containing anthocyanin-related genes and metabolites based on transcriptome and metabolome analysis (Figure 6). The expression and production levels of DEGs and DPMs between GB767 and PB767 are labeled in different colors. In the early stage of anthocyanin biosynthesis, the expression levels of 4 *PAL*, 3 *C4H*, and 1 *CHS* in PB767 were significantly higher than that in GB767, but there was no difference in the accumulation of early metabolites, such as chalcone and naringenin. The gene *F3′H* (*BolC09g064000.2J*) could catalyze the reaction from dihydrokaempferol to dihydroquercetin, and its expression level in PB767 was significantly higher than that in GB767, which is consistent with dihydrokaempferol in PB767 being significantly lower than that in GB767. In the late stage of anthocyanin biosynthesis, the production level of cyanidin-3-O-sophorotrioside, delphinidin-3-O-glucoside, delphinidin-3-O-galactoside, and petunidin-3-O-glucoside in PB767 was significantly higher than that in GB767. In particular, delphinidin-3-O-glucoside, delphinidin-3-O-galactoside, and cyanidin-3-O-sophorotrioside accounted for 80.5% of all detected anthocyanins and were the three major anthocyanins in PB767. However, cyanidin-3-O-glucoside, cyanidin-3-O-galactoside, and pelargonidin-3-O-glucoside had a higher production level in GB767 than PB767. Among them, cyanidin-3-O-glucoside and cyanidin-3-O-galactoside accounted for 96.5% of all detected anthocyanins in GB767. The total content of modified anthocyanins detected in PB767 was three times that in GB767, which was consistent with the higher expression level of *UFGT* (*BolC02g007850.2J*) in PB767 than in GB767. 

**Figure 7:**
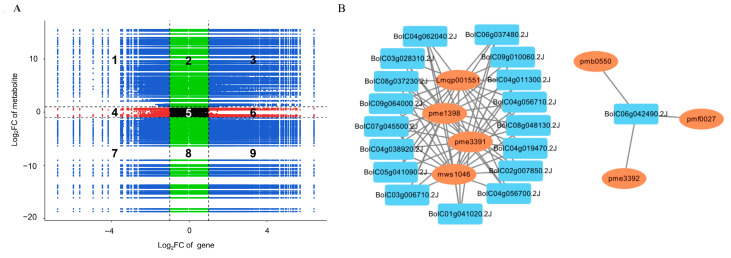
Correlation analysis of anthocyanin-related genes and metabolites. (**A**) Nine-quadrant plot. (**B**) Correlations between anthocyanin-related genes and metabolites.

To understand the relationship between anthocyanin-related genes and metabolites, we performed a correlation analysis on them. In the nine-quadrant plot, genes and metabolites in the third and seventh quadrants showed consistent changes (Figure 7A). In total, 2979 genes and 171 metabolites that had Pearson correlation coefficient values ≥0.8 were identified in the 3rd and 7th quadrants, which indicated that differences in these metabolites may be positively regulated by these genes. Among these genes and metabolites, 18 anthocyanin-related genes were identified and showed positive correlations with seven modified anthocyanins (Figure 7B). For example, the expression levels of *PAL* (*BolC04g011300.2J*, *BolC04g062040.2J*, *BolC08g037230.2J,* and *BolC04g038920.2J*), *C4H* (*BolC04g019470.2J*, *BolC04g056700.2J,* and *BolC04g056710.2J*), *CHS* (*BolC03g006710.2J*), *F3′H* (*BolC09g064000.2J*), *UFGT* (*BolC02g007850.2J*), *AT* (*BolC08g048130.2J*), *UGT* (*BolC07g045500.2J*, *BolC05g041090.2J,* and *BolC01g041020.2J*), *MYB* (*BolC03g028310.2J* and *BolC06g037480.2J*), and *LBD* (*BolC09g010060.2J*) were positively correlated with the content of cyanidin-3-O-sophorotrioside (lmqp001551), delphinidin-3-O-glucoside (pme1398), delphinidin-3-O-galactoside (mws1046), and petunidin-3-O-glucoside (pme3391). The contents of cyanidin-3-O-glucoside (pmb0550), cyanidin-3-O-galactoside (pmf0027), and pelargonidin-3-O-glucoside (pme3392) were positively correlated with *MYBL2.1* (*BolC06g042490.2J*).

## 3. Discussion

### 3.1. Delphinidin-Based Anthocyanins in the Floret Sepals Are Responsible for Purplish Curds in PB767 

Anthocyanins such as cyanidin, pelargonidin, and delphinidin can produce various tones of colors ranging from pale yellow to deep violet in the vegetative or floral organs of plants [27]. Generally speaking, plant organs with blue-purple colors tend to contain delphinidin-based anthocyanins, red-purple organs contain predominantly cyanidin-based anthocyanins, and orange-red organs contain pelargonidin-based anthocyanins [28]. It is not surprising that anthocyanidin accumulation, due to the natural mutations in those genes involved in the cyanin biosynthetic pathways, leads to purple colors in specific organs [29]. In most *Brassica* species such as cabbage (*B. oleracea* L. var. *capitate*), Chinese cabbage (*B. rapa* ssp. *pekinensis* L.), and kale (*B. oleracea* L. var. *acephala*), their purple colors in leaf and other organs are mainly achieved by the accumulation of cyanidin-based anthocyanins [30,31,32,33]. Similarly, in purple cauliflower (*B. oleracea* L. var. *botrytis*), cyanidin is also the predominant anthocyanin [34]. A recent investigation in purple broccoli reported the presence of cyanidin, delphinidin, and malvidin in the flower buds, suggesting that they might contribute to the formation of purple color in broccoli [35]. However, due to the lack of quantitative experiments, it remains to be determined whether broccoli also uses cyanidin-based anthocyanins to produce purple curd. 

In the present study, the widely targeted detection of flavonoid metabolome identified eight types of anthocyanins, and seven were differentially accumulated in the sepal samples between GB767 and PB767 NILs. As previously expected, the total anthocyanin content of the sepal in PB767 was more than three times higher than that of GB767, accounting for the purple curds of PB767. As pelargonidin-based anthocyanins tend to yield orange to red colors, we, therefore, dwell on the changes in the cyanidin- and delphinidin-based anthocyanins. First, the total content of the cyanidins and delphinidins that probably confer purplish sepals was compared between NILs. Interestingly, cyanidins accounted for 97.7% of anthocyanin in GB767, suggesting that cyanidin is the dominant anthocyanin in green curds. On the contrary, 71.6% of anthocyanins were delphinidins and their derivates in PB767, indicating that delphinidin-based anthocyanins were the major anthocyanin in purple curds. To the best of our knowledge, these findings for the first time reveal the presence of delphinidins in purple broccoli, which is unusual for purple Brassica vegetables.

With respect to the cyanidins, the content of cyanidin-3-O-sophorotrioside increased by 53.4-fold in purple curd, while the content of other cyanidins, cyanidin-3-O-galactoside, and cyanidin-3-O-sophorotrioside, decreased 3.3- and 8.9-fold, respectively. As such, the total cyanidin content was comparable between GB767 and PB767 NILs. By contrast, the content of delphinidins and their derivates (delphinidin-3-O-glucoside, delphinidin-3-O-galactoside, and petunidin-3-O-glucoside) was 170.8 times higher than that of GB767. Considering that delphinidins were the major anthocyanins in PB767, we concluded that delphinidin-based anthocyanins might be responsible for the blue-purple colors of PB767 curds. Taken together, these results suggest that, unlike other *Brassica* crops, delphinidin is the major anthocyanin, which leads to the purple sepals of flower buds and finally the purple curds in PB767.

### 3.2. One Broccoli CYP75B/F3′H Might Acquire Flavonoid 5′-Hydroxylase Activities, Promoting the Accumulation of Delphinidins in Purple Curds

The hydroxylation reactions from dihydrokaempferol to cyanidin and delphinidin are catalyzed by two cytochromes P450 monooxygenases (CYP), flavonoid 3′-hydroxylase (F3′H/CYP75B) and flavonoid 3′,5′-hydroxylase (F3′5′H/CYP75A), respectively [36,37]. It is reported that some plant species lack purple/violet colors due to the absence of *CYP75A* (F3′5′H) genes in their genomes and, therefore, are unable to biosynthesize delphinidins. For example, *Rosa hybrida* does not possess the F3′5′H/CYP75A enzyme, resulting in the lack of violet to blue flower varieties in nature [38]. *CYP75A* genes were also absent in many Brassica species such as *Arabidopsis thaliana*, *B. rapa*, and *B. napus* as well as *B. oleracea*, indicating that they might have lost the *CYP75A* subfamily during Brassica evolution and should theoretically be unable to produce delphinidin-based anthocyanidins. Accordingly, *F3′5′H* was not found in the transcriptome analysis; however, a huge amount of delphinidin-based cyanidins were detected in PB767 curds. Surprisingly, not limited to purple broccoli, delphinidins have been detected in purple non-heading Chinese cabbage (*B. rapa*), purple caitai (*B. compestris* var *tsai-tai* Hort), and purple cauliflower (*B. oleracea* var *botrytis*) [35,39,40]. Although delphinidins might not be the major anthocyanins among those vegetables, their observations demonstrated that delphinidins could also be produced in specific species. So far, there are no genes encoding F3′5′H enzymes in these Brassica genomes. The contradicting facts between genome (transcriptome) and metabolome data lead to the notion that other enzyme(s) instead of F3′5′H could catalyze the hydroxylation reactions to delphinidin. 

Both F3′H and F3′5′H belong to the CYP75 family, and they can be further classified into two subfamilies, CYP75B and CYP75A, despite the nearly 50% sequence similarity between the two subfamilies [41,42]. Despite the high sequence similarity between the CYP75B/F3′H and CYP75A/F3′5′H subfamilies, their divergence predated early plant evolution [41]. As a consequence, the distribution of *CYP75A/F3′5′H* is scattered among plant genomes, while *CYP75B*/*F3′H* exhibits a ubiquitous presence in higher plants [41,43]. In the Asteraceae genus, three *CYP75B*s encoding F3′H according to their sequences exhibited F3′5′H activity [41], and subsequent studies demonstrated that the alternated substrate-binding specificity of Asteraceae CYP75B/F3′H is mainly attributed to the substitution of a conserved amino acid located in the substrate recognition site 6 (SRS6) [44]. Surprisingly, a Thr to Ser/Ala exchange at the 8th amino acid of SRS6 can confer additional 5′-hydroxylation activity to traditional F3′H enzymes [44]. In our transcriptome analysis, one *CYP75B/BolC09g064000.2J* was among the DEGs that exhibited much higher expressions in the PB767 lines compared with GB767, and qRT-PCR experiments confirmed that the expression level of *CYP75B/BolC09g064000.2J* was significantly higher in sepals of PB767. Consistent with an earlier study in broccoli [45], we also identified a 43 bp deletion in the 2nd exon of *CYP75B/BolC09g064000.2J* in GB767, resulting in an F3′H transcript with a premature stop codon. The nonsense transcripts of F3′H could lead to the reduction in cyanidins, and accordingly, the content of cyanidin-3-O-sophorotrioside in the curds, compared with PB767, decreased dramatically in GB767. Likewise, the expression of *CYP75B/BolC09g064000.2J* was significantly higher in PB767 curds, indicating that *BolC09g064000.2J* might be involved in the cyanidin biosynthesis. 

Meanwhile, an extremely high level of delphinidin accumulation was observed in purple curds. Given that the Asteraceae CYP75Bs acquire 5′-hydroxylase activity, it is tempting to speculate that *CYP75B/BolC09g064000.2J*, the only differential expressed F3′H between NILs, might have experienced a similar evolution process, therefore enabling the biosynthesis of delphinidins in purple curds. Unfortunately, sequence alignments did not identify any nonsynonymous mutations in the SRS6, as well as other regions of *CYP75B/BolC09g064000.2J* in PB767, which is in agreement with its role in cyanidin biosynthesis. Thus, it is likely that other *CYP75B*-like genes function as 5′-hydroxylase during delphinidin biosynthesis. We comprehensively inspected our transcriptome data set for the occurrence of non-canonical CYP75Bs as observed in the Asteraceae genus. In this regard, phylogenetic analysis was performed based on the CYPs that were significantly higher expressed in PB767, and four non-redundant CYP75B-likes genes in the same subclade were identified according to their phylogenetic relationships (Figure S4). Notably, a substitution from Thr to Ala at the 8th amino acid of SRS6 was identified with one *F3′H* (*BolC02g015480.2J*), implying it might be a non-canonical *F3′H* gene catalyzing the hydroxylation reactions associated with delphinidin biosynthesis (Figure S4). Further work will be focused on investigating the enzymatic property of the *CYP75B*/*BolC07g026840.2J* and elucidating its potential mechanistic basis. 

### 3.3. Anthocyanin Biosynthesis-Related Genes Are Differentially Regulated in Purple Broccoli 

Transcriptome sequencing suggested 19 anthocyanin biosynthesis-related genes were differentially expressed between the NILs. Among them, fifteen were structural genes, and four genes encode transcription factors. At the early stage of anthocyanin biosynthesis, the *PALs* (*BolC04g011300.2J*, *BolC04g062040.2J*, *BolC08g037230.2J,* and *BolC04g038920.2J*), *C4Hs* (*BolC04g019470.2J*, *BolC04g056700.2J,* and *BolC04g056710.2J*), and *CHS* (*BolC03g006710.2J*) showed higher expression levels in PB767 than that in GB767, which was consistent with the previous findings in purple Chinese cabbage. Consistently, *BolMYB12.2* (*BolC03g028310.2J*), controlling the expressions of *PAL*, *C4H*, and *CHS,* was also significantly higher expressed in PB767. The high expression of these EBGs could facilitate the production of precursor substrates for anthocyanin in PB767. On the other hand, some LBGs including *CYP75B/F3′H*, *UFGT*, *AT,* and *UGT,* also showed higher expression levels in PB767. As discussed above, we speculated that the *CYP75B/F3′H* probably encodes non-canonical flavonoid 3′-hydroxylase which might have 5′-hydroxylase activity. One *UFGT* gene (*BolC02g007850.2J*), three *UGT* genes (*BolC07g045500.2J*, *BolC05g041090.2J,* and *BolC01g041020.2J*), and one *AT* gene (*BolC08g048130.2J*) were higher expressed in PB767 than in GB767. The increased levels of EBG and LBG expressions contributed to anthocyanin biosynthesis.

From the aspect of TFs, the MYBs act as critical transcriptional regulators in the process of anthocyanin biosynthesis [46,47]. In *Arabidopsis*, MYB11 and MYB12 independently regulated the expressions of the EBGs [48]. MYBs can interact with bHLH and WD40 to form an MBW protein complex, and then activate the LBGs such as *F3′H*, *DFR,* and *ANS*. In this study, the expressions of *MYB12.2* (*BolC03g028310.2J*) and *MYB2* (*BolC06g037480.2J*) were upregulated, while *BolMYBL2.1* (*BolC06g042490.2J*) was downregulated in PB767. The expression behaviors of these MYBs were similar to that of their *Arabidopsis* counterparts. In addition, some TFs, such as WRKYs, AP2/ERF-ERFs, and MADSs, displayed significantly different expression patterns between NILs, indicating their possible roles in regulating EBGs and LBGs in the anthocyanin biosynthetic pathways. Thus, the results showed that anthocyanin biosynthesis and accumulation are probably influenced by a series of complicated processes in broccoli.

## 4. Materials and Methods

### 4.1. Plant Materials and Sample Preparation

The curds of a pair of near-isogenic lines, GB767 and PB767, from an experimental field at the Sijiqing Farm of the Beijing Academy of Agriculture and Forestry Sciences were used in this study. Nearly isogenic lines (NILs) that differ in curd colors were developed from crosses between two inbred lines, XXXX (purple-curd) and XXX (green-curd). Then, six rounds of backcrossing with green-curd parent were carried out to ensure that PB767 and GB767 exhibit the same agronomic traits except for curd colors. The outer sepals of the flower buds were collected from three plants each of GB767 and PB767 at the same time in spring. After labeling, they were put into 2 mL centrifuge tubes and immediately frozen with liquid nitrogen. The frozen samples were stored at −80 °C for further transcriptome and metabolome analysis.

### 4.2. RNA Library Preparation and Transcriptome Sequencing Analysis

After extracting total RNA and digesting DNA with DNase, a total amount of 3 μg RNA per sample was used for the RNA library preparations. Eukaryotic mRNA was enriched with poly-T oligo-attached magnetic beads. First-strand cDNA was synthesized with six base random primers and M-MuLV Reverse Transcriptase using the interrupted mRNA as a template. Second-strand cDNA synthesis was subsequently performed using DNA Polymerase I and RNase H. The purified double-strand cDNA was subjected to terminal repair (3′ ends plus A base), and then connected with a sequencing connector and PCR amplification was carried out. The library quality was assessed on the Bioanalyzer 2100 system (Agilent Technologies, Santa Clara, CA, USA). Thereafter, the library preparations were sequenced on an Illumina Hiseq platform and 125 bp/150 bp paired-end reads were generated. After preprocessing the raw data, the filtered reads were mapped to the reference genome of cabbage (*Brassica oleracea* L. var. *capitata*) using the HISAT2 software (Version 2.2.1) [49]. The fragments per kilobase of transcript per million mapped reads (FPKM) method was used to calculate the mRNA expression level. Differential expression analysis between the two groups of colored samples was performed using the DESeq2 R package [50]. Finally, differential mRNA was screened by the following parameters: |log2foldchange| ≥ 1 and the adjusted *p* value < 0.05. Gene Ontology (GO) and Kyoto Encyclopedia of Genes and Genomes (KEGG) enrichment analyses of differential mRNA were implemented by the GOSeq R package and the KOBAS software (Version 3.0), respectively [51,52]. The transcriptome sequencing was conducted by the Biomarker Technologies Co., Ltd. (Beijing, China).

### 4.3. Validation of RNA-Seq Data by qRT-PCR

Total RNA was extracted using TransZol Kit (TransGen Biotech Co., Ltd., Beijing, China) with modifications [53]. The first-strand cDNA was synthesized using HiScript III RT SuperMix for qPCR (Vazyme Biotech Co., Ltd., Beijing, China). The qRT-PCR analyses were performed on a fluorescence quantification instrument (Roche LightCycler 480 fluorescence quantification system, Indianapolis, IN, USA), and the enzyme and fluorescent dye were provided by Taq Pro Universal SYBR qPCR Master Mix (Vazyme Biotech Co., Ltd., Beijing, China). The gene-specific primers are shown in Table S7. The 2^−ΔΔCT^ method was used to calculate the relative expression levels [54]. The reaction system (20 μL) contained 1 μL cDNA template, 1 μL gene-specific primers (0.5 μL forward primer and 0.5 μL reverse primer), 10 μL 2 × Taq Pro Universal SYBR qPCR Master Mix, and 8 μL ddH_2_O. The parameters of the reaction procedure were set as follows: 95 °C for 30 s, followed by 40 cycles of 95 °C for 10 s, 60 °C for 10 s, and then 72 °C for 10 s.

### 4.4. Metabolite Extraction and UPLC Conditions

Freeze-dried sepals stored at −80 °C were crushed using a mixer mill (Retsch GmbH MixMill 400, Germany) with a zirconia bead for 15 min at 30 Hz. Then, 100 mg of each powder sample was weighed and extracted overnight at 4 °C with 1.0 mL 70% aqueous methanol. Following centrifugation at 10,000× *g* for 10 min, the supernatant was removed and filtered (SCAA-104, 0.22 μm pore size; ANPEL, Shanghai, China) before the UPLC-MS/MS analysis. The sample extracts were analyzed using a UPLC-ESI-MS/MS system (UPLC, Shim-pack UPLC Shimadzu CBM30A system, Shanghai, China; MS, Applied Biosystems 6500 Q TRAP, Shanghai, China). The samples were analyzed under the following HPLC conditions: column, Waters ACQUITY UPLC HSS T3 C18 (1.8 µm, 2.1 mm × 100 mm); solvent system, water (0.04% acetic acid): acetonitrile (0.04% acetic acid); gradient program, 100:0 *v*/*v* at 0.0 min, 5:95 *v*/*v* at 11.0 min, 5:95 *v*/*v* at 12.0 min, 95:5 *v*/*v* at 12.1 min, and 95:5 *v*/*v* at 15.0 min; flow rate, 0.40 mL/min; temperature, 40°C; and injection volume: 2 µL. The effluent was alternatively connected to an ESI-triple quadrupole-linear ion trap (Q TRAP)-MS. The identification of metabolic compounds were determined by comparing the flight time with the standard substance.

### 4.5. Qualitative and Quantitative Analysis of Metabolites

The quantitative detection of metabolites between GB767 and PB767 was performed using multiple reaction monitoring (MRM) by the MetWare Biotechnology Co., Ltd. (Wuhan, China). Linear ion trap (LIT) and triple quadrupole (QQQ) scans were acquired on an API 6500 Q TRAP UPLC/MS/MS system, which was equipped with an ESI Turbo Ion-Spray interface. The system was controlled by Analyst 1.6.3 software (AB Sciex, Concord, ON, Canada) and operated in positive ion mode. The operational parameters of ESI were as follows: ion source, turbo spray; source temperature, 550 °C; ion spray voltage (IS), 5500 V; ion source gas I (GSI), gas II (GSII), curtain gas (CUR), 55 psi, 60 psi, and 25.0 psi, respectively; and collision gas (CAD), high. Instrument tuning and mass calibration were performed with 10 μmol/L and 100 μmol/L polypropylene glycol solutions in the QQQ and LIT modes, respectively. The QQQ scans were acquired via the MRM experiments with the collision gas (nitrogen) set to 5 psi. DP and CE for individual MRM transitions were performed with further DP and CE optimization. A specific set of MRM transitions was monitored for every period according to the metabolites eluted within this period [55].

The overall difference in metabolic profiles and differentially produced metabolites (DPMs) between GB767 and PB767 were analyzed using three statistical analysis methods (PCA, PLS-DA, and OPLS-DA). The metabolites with variable importance in projection (VIP) ≥ 1 and |log2foldchange| ≥ 1 were considered differential metabolites. The KEGG pathway enrichment analysis of DPMs was performed using the hypergeometric test to identify the pathway items that were significantly enriched. The threshold of the p value was set to 0.05.

### 4.6. Correlation Analysis between Transcriptome and Metabolome Data

Pearson correlation coefficients were calculated to integrate transcriptome and metabolome data according to previous described method [56]. In this study, the data were uniformly log-converted before analysis. For the joint analysis between the transcriptome and metabolome, the screening criterion was a Pearson correlation coefficient great than 0.8.

### 4.7. Statistical Analysis

Each experiment was set up with three biological replicates, and data were expressed as the mean ± standard deviation (SD). The results of the qRT-PCR analysis were statistically compared by *t*-test using the SPSS.21 software.

## 5. Conclusions

In this study, phenylpropanoid and flavonoid biosynthesis pathways were enriched through metabolic analyses, which is corroborated by the fact that total anthocyanin contents are much higher in the floret sepals of PB767 broccoli. Further analysis suggested that delphinidins are predominant anthocyanins in the broccoli cultivar used in this experiment. To the best of our knowledge, this is the first report that delphinidins (delphinidin-3-O-glucoside and delphinidin-3-O-galactoside) are major anthocyanins in the broccoli cultivar PB767. Transcriptome analysis indicated that many genes associated with the anthocyanin biosynthetic pathways were highly expressed in the purple broccoli. Consistently, the expressions of several transcriptional regulators, such as MYBs, LBDs, and ERFs, were upregulated in the purple broccoli. Interestingly, we also found that one non-canonical *BolCYP75B*, *BolC02g015480.2*, with significantly higher expression in purple curds could be involved in the production of delphinidins in PB767. Correlation analysis further suggested that the identified 19 genes were positively correlated with seven differentially accumulated anthocyanins in purple broccoli. Overall, the results identified the structural and regulatory genes that are responsible for anthocyanin biosynthesis, which provides a theoretical basis for the molecular breeding of broccoli varieties with high anthocyanin content.

## Figures and Tables

**Table 1:** Summary of the transcriptome sequencing data quality of six samples.

Samples	Clean Reads	Clean Bases	Q30	Rate of GC Content
GB767-1	21.11 Mb	6.32 Gb	93.98%	47.73%
GB767-2	20.97 Mb	6.36 Gb	93.91%	47.48%
GB767-3	20.85 Mb	6.23 Gb	93.96%	47.49%
PB767-1	26.79 Mb	8.02 Gb	94.41%	47.07%
PB767-2	22.29 Mb	6.67 Gb	94.00%	47.34%
PB767-3	23.70 Mb	7.08 Gb	93.83%	47.39%

**Table 2:** Differentially produced intermediate metabolites in anthocyanin-related pathways between GB767 and PB767.

Compounds	GB767	PB767	Log2FC	Regulated	KEGG Map
3-O-Acetylpinobanksin	-	2.00 × 10^4^	11.12	up	ko00941
Aromadendrin	8.85 × 10^3^	-	−9.94	down	ko00941, ko01100, ko01110
Gallocatechin	-	2.71 × 10^4^	11.56	up	ko00941, ko01110
Pelargonidin-3-O-glucoside	2.59 × 10^5^	-	−14.82	down	ko00942, ko01100, ko01110
Delphinidin-3-O-glucoside	1.58 × 10^5^	2.28 × 10^7^	7.17	up	ko00942
Cyanidin-3-O-glucoside	1.91 × 10^7^	5.74 × 10^6^	−1.73	down	ko00942
Petunidin-3-O-glucoside	1.25 × 10^4^	8.92 × 10^6^	9.47	up	ko00942
2,6,7,4’-Tetrahydroxyisoflavanone	1.20 × 10^5^	1.63 × 10^4^	−2.87	down	ko00943, ko01110
Quercetin-3-O-sophoroside	2.64 × 10^5^	3.50 × 10^7^	7.05	up	ko00944
Kaempferol-3-O-sophorotrioside	6.73 × 10^6^	1.29 × 10^6^	−2.38	down	ko00944
Kaempferol-3-O-galactoside	2.95 × 10^5^	2.80 × 10^4^	−3.40	down	ko00944
Quercetin-3-O-glucoside	7.85 × 10^4^	5.69 × 10^5^	2.86	up	ko00944, ko01100, ko01110
Apigenin-7-O-glucoside	1.34 × 10^5^	-	−13.86	down	ko00944
Kaempferol-3-O-glucoside	5.73 × 10^6^	4.78 × 10^5^	−3.58	down	ko00944, ko01110
Kaempferol-3-O-sophoroside	3.00 × 10^5^	7.62 × 10^4^	−1.98	down	ko00944
Luteolin-7-O-glucoside	3.85 × 10^6^	-	−18.71	down	ko00944

**Note:** “-” indicates that this compound was not detected in the sample.

**Table 3:** The differentially produced anthocyanins in GB767 and PB767.

Categories	Metabolites	GB767	PB767	Log2FC	Regulated
Cyanidin	Cyanidin-3-O-sophorotrioside	2.96 × 10^5^	1.58 × 10^7^	5.74	up
	Cyanidin-3-O-glucoside	1.91 × 10^7^	5.74 × 10^6^	−1.73	down
	Cyanidin-3-O-galactoside	5.58 × 10^6^	6.28 × 10^5^	−3.15	down
Pelargonidin	Pelargonidin-3-O-glucoside	2.59 × 10^5^	-	-	down
Delphinidin	Delphinidin-3-O-glucoside	1.58 × 10^5^	2.28 × 10^7^	7.17	up
	Delphinidin-3-O-galactoside	1.58 × 10^5^	2.44 × 10^7^	7.27	up
Petunidin	Petunidin-3-O-glucoside	1.25 × 10^4^	8.92 × 10^6^	9.47	up

**Note:** “-” indicates that this compound was not detected in the sample.

## Data Availability

The data presented in this study are available on request from the corresponding authors. RNA-seq raw data were deposited in the NCBI Sequence Read Archive (SAR) database (BioProject ID PRJNA904066).

## Supplementary Materials

The following supporting information can be downloaded at: https://www.mdpi.com/article/10.3390/ijms24076115/s1.
