# Supplementary material for: Comparative Transcriptome and Metabolome Analyses of Broccoli Germplasms with Purple and Green Curds Reveal the Structural Genes and Transitional Regulators Regulating Color Formation
Source: Int J Mol Sci. 2023 Mar 24;24(7):6115. doi: 10.3390/ijms24076115 (PMC10094742; doi:10.3390/ijms24076115)
Supplement: Supplementary file 1 [file ijms-24-06115-s001.zip › Supplementary_Figures.pdf]

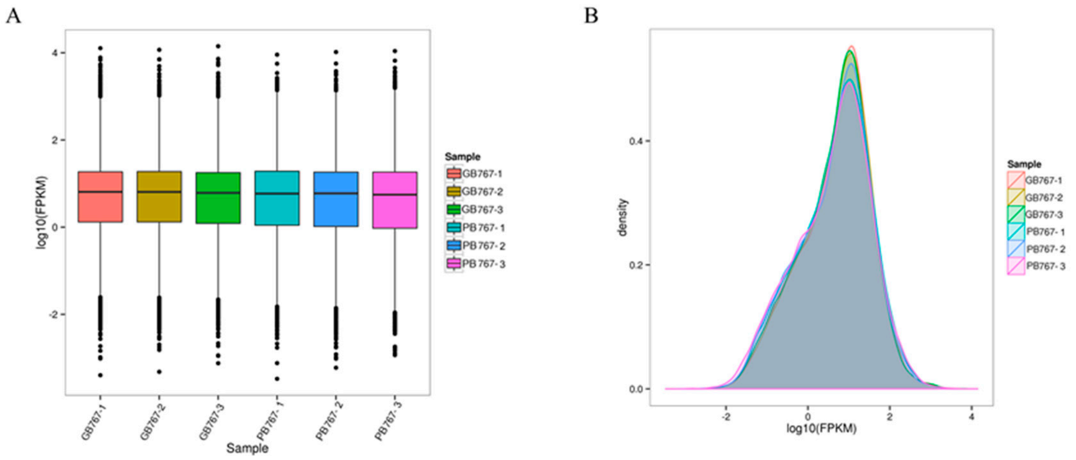

Figure S1: Box plot (A) and density plot (B) of FPKM for GB767 and PB767 of six samples.

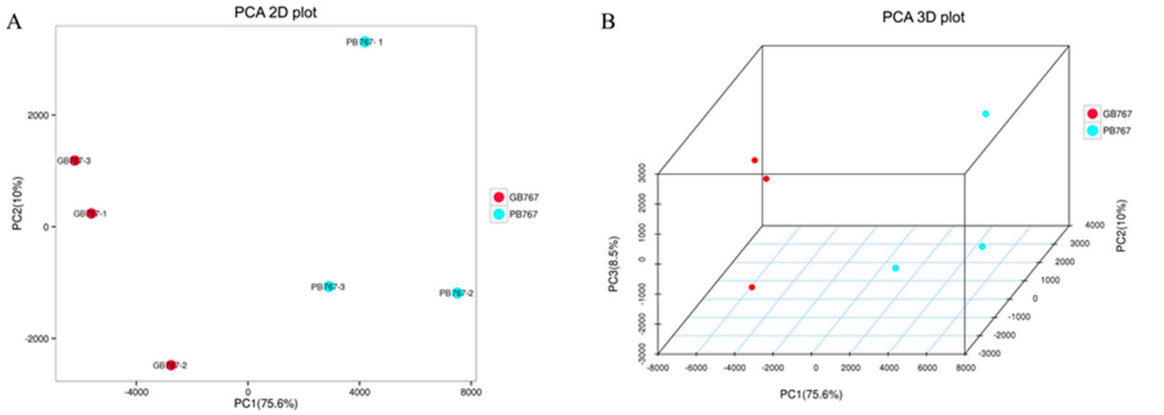

Figure S2: PCA analysis of gene expression in six samples of GB767 and PB767. (A) PCA 2D plot. (B) PCA 3D plot.

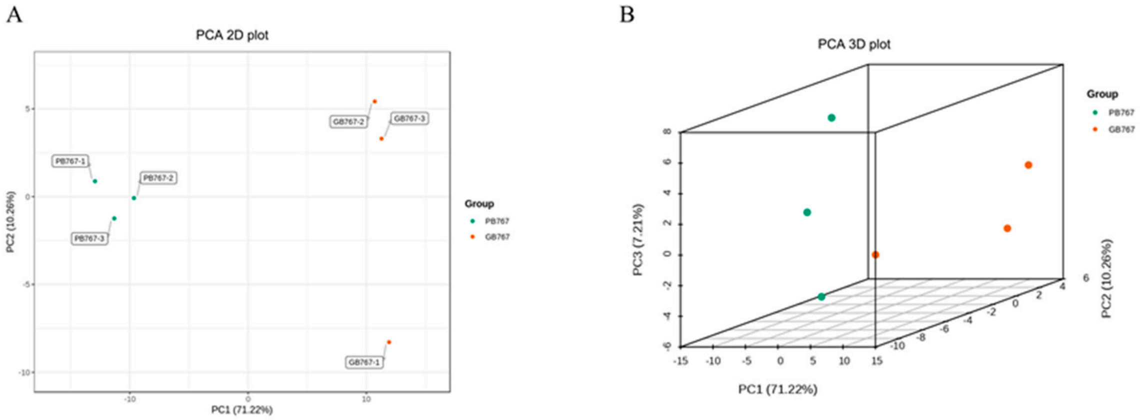

Figure S3: PCA analysis of metabolite content in 6 samples of GB767 and PB767. (A) PCA 2D plot. (B) PCA 3D plot.

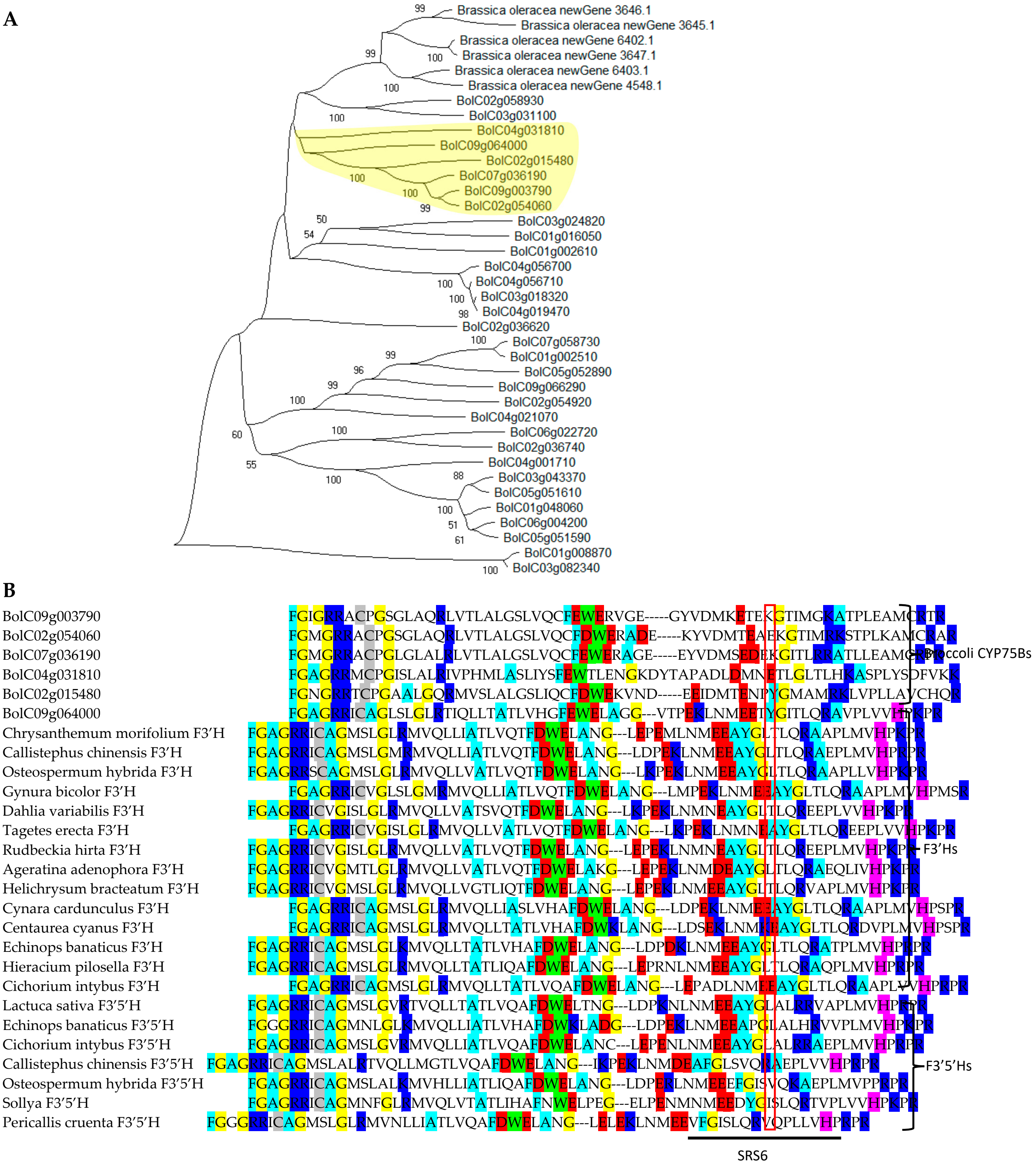

Figure S4. Analysis of CYP75Bs that exhibited higher expressions in PB767. (A) Neighbor-joint tree was constructed based on these broccoli CYPs with higher expressions in purple curds. The clade of broccoli CYP75Bs were highlighted in yellow. (B) Sequence alignment of selected F3′H, F3′5′H, and broccoli CYP75Bs. The amino acid sequences are shadowed with colors based on the sequence similarity. The protein sequences of F3′H and F3′H-derivated F3′5′H were adopted from Seitz et. al. (2015). The 8th position of the amino acid of substrate recognition site 6 (SRS6) was boxed in red.
